# Survival Comes at a Cost: A Coevolution of Phage and Its Host Leads to Phage Resistance and Antibiotic Sensitivity of *Pseudomonas aeruginosa* Multidrug Resistant Strains

**DOI:** 10.3389/fmicb.2021.783722

**Published:** 2021-12-02

**Authors:** Sarshad Koderi Valappil, Prateek Shetty, Zoltán Deim, Gabriella Terhes, Edit Urbán, Sándor Váczi, Roland Patai, Tamás Polgár, Botond Zsombor Pertics, György Schneider, Tamás Kovács, Gábor Rákhely

**Affiliations:** ^1^Department of Biotechnology, University of Szeged, Szeged, Hungary; ^2^Institute of Plant Biology, Biological Research Center, Szeged, Hungary; ^3^Doctoral School of Biology, University of Szeged, Szeged, Hungary; ^4^Department of Clinical Microbiology, University of Szeged, Szeged, Hungary; ^5^Department of Pathophysiology, Faculty of Medicine, University of Szeged, Szeged, Hungary; ^6^Institute of Biophysics, Biological Research Center, Szeged, Hungary; ^7^Doctoral School of Theoretical Medicine, University of Szeged, Szeged, Hungary; ^8^Department of Medical Microbiology and Immunology, University of Pécs, Pécs, Hungary; ^9^Department of Biotechnology, Nanophagetherapy Center, Enviroinvest Corp., Pécs, Hungary; ^10^Biopesticide Ltd., Pécs, Hungary

**Keywords:** bacteriophage therapy, combined treatment, phage resistance, phage-provoked sequential genomic mutation/deletion, MexXY-OprM efflux system

## Abstract

The increasing ineffectiveness of traditional antibiotics and the rise of multidrug resistant (MDR) bacteria have necessitated the revival of bacteriophage (phage) therapy. However, bacteria might also evolve resistance against phages. Phages and their bacterial hosts coexist in nature, resulting in a continuous coevolutionary competition for survival. We have isolated several clinical strains of *Pseudomonas aeruginosa* and phages that infect them. Among these, the PIAS (Phage Induced Antibiotic Sensitivity) phage belonging to the *Myoviridae* family can induce multistep genomic deletion in drug-resistant clinical strains of *P. aeruginosa*, producing a compromised drug efflux system in the bacterial host. We identified two types of mutant lines in the process: green mutants with SNPs (single nucleotide polymorphisms) and smaller deletions and brown mutants with large (∼250 kbp) genomic deletion. We demonstrated that PIAS used the MexXY-OprM system to initiate the infection. *P. aeruginosa* clogged PIAS phage infection by either modifying or deleting these receptors. The green mutant gaining phage resistance by SNPs could be overcome by evolved PIASs (E-PIASs) with a mutation in its tail-fiber protein. Characterization of the mutant phages will provide a deeper understanding of phage-host interaction. The coevolutionary process continued with large deletions in the same regions of the bacterial genomes to block the (E-)PIAS infection. These mutants gained phage resistance *via* either complete loss or substantial modifications of the phage receptor, MexXY-OprM, negating its essential role in antibiotic resistance. *In vitro* and *in vivo* studies indicated that combined use of PIAS and antibiotics could effectively inhibit *P. aeruginosa* growth. The phage can either eradicate bacteria or induce antibiotic sensitivity in MDR-resistant clinical strains. We have explored the potential use of combination therapy as an alternative approach against MDR *P. aeruginosa* infection.

## Introduction

*Pseudomonas aeruginosa* is one of the most lethal causative organisms of bacteremia. A recent study showed that patients with *Pseudomonas* bloodstream infection had a higher mortality rate than those infected by the members of *Enterobacteriaceae* (Shi et al., 2019). Moreover, *P*. *aeruginosa* has intrinsically evolved resistance to “drugs of last resort,” leading to emergent strains that are pan-drug-resistant (Magiorakos et al., 2012; Sid Ahmed et al., 2019). The WHO has published a list of multidrug-resistant (MDR) bacterial pathogens of global significance, prioritizing *P. aeruginosa* as a critical worldwide public health threat (World Health Organization [WHO], 2019). *P. aeruginosa* commonly occurs in the environment and is dispersed readily among healthy individuals. However, in immune-compromised hosts it can cause invasive and hostile infections such as acute pneumonia or bloodstream infection. In addition, *P. aeruginosa* can seriously harm cystic fibrosis (CF) patients and increase morbidity and mortality (Malhotra et al., 2019). The primary complications in CF patients begin with chronic infection of the airways caused predominantly by *P*. *aeruginosa*. This infection is commonly treated by antibiotics. The extensive use of antibiotics to treat *P. aeruginosa* has produced many antibiotic-resistant strains. The MDR *P. aeruginosa* infection in CF patients can foster chronic biofilm lung infections, making it extremely difficult to eradicate. The genomes of *P. aeruginosa* strains have almost 6000 genes, facilitating ecological flexibility. The increased number of genes in MDR strains enables *P. aeruginosa* to develop resistance to almost all current antipseudomonal agents through chromosomal mutations (López-Causapé et al., 2018a; Druge et al., 2019). The most common underlying mechanisms of drug resistance in *P. aeruginosa* are porin channel alterations and/or modified efflux pumps *via* either SNPs or overexpressing the pump (Morita et al., 2012; Frimodt-Møller et al., 2018; López-Causapé et al., 2018b; Eichenberger and Thaden, 2019). The lack of new antibiotics and the increasing ineffectiveness of conventional antibiotics have exacerbated MDR resistance. Thus, the need for novel therapeutic approaches is urgent. Bacteriophage (phage) therapy represents a promising alternative solution to combat MDR *P. aeruginosa* strains (Roach and Debarbieux, 2017).

Phage-based therapy exploits the ability of phages to infect target bacterial hosts selectively. Their bactericidal activity is precise in infecting and killing only a particular host with low inherent toxicity. Phages can be applied alone, in phage cocktails, or combined with drugs. However, as preclinical and clinical studies are limited, phage therapy is yet to realize its full potential as an antibacterial approach. Advances in molecular biological techniques have opened a wide range of genetic techniques that have revolutionized how we view phage therapy today. A recent clinical trial with an engineered phage cocktail against multidrug *Mycobacterium abscessus* in CF patients is reassuring (Dedrick et al., 2019). Some recent *in vivo* phage therapy approaches have successfully treated MDR *P. aeruginosa* infections in mice by administering either a spray-dried phage formulation (Chan et al., 2018) or a cocktail of multiple broad range phages (Forti et al., 2018) or phages combined with other drugs.

However, the bacterial host is not entirely defenseless against infecting phages. Bacteria and bacteriophages coexist in nature through continuous coevolution for survival (Labrie et al., 2010; Samson et al., 2013). This reciprocal adaptation and counter-adaptation in ecologically interacting species are modulated *via* various defense mechanisms. The most common strategies include memorizing previous infections by CRISPR–Cas systems (Marraffini, 2015), which might be overcome by anti-CRISPR phage genes that hinder the Cas system (Rauch et al., 2017; Stanley et al., 2019); bacterial surface receptor modifications preventing adsorption of phages (Holst Sørensen et al., 2012) versus mutation in the phage genome to overcome the bacterial surface modification (Kim and Ryu, 2012); restriction–modification systems that degrade unmodified foreign DNA (Oliveira et al., 2014) versus anti-restriction strategies of phages (Ravin et al., 2002); and metabolic arrest caused by bacterial abortive infection systems (Dy et al., 2014), which was being circumvented by phages through *motA* gene mutation (Hinton, 2010). These continual evolutionary battles can also lead to the emergence of changes in their genomes (Tanji et al., 2008; Le et al., 2014). The primary contact of the phage with the bacterial surface is the first line of bacterial defense. Phages initiate infection by binding phage to the surfaces of bacteria (Rakhuba et al., 2010). The most studied phage receptors in Gram-negative bacteria are lipopolysaccharide (LPS) moieties present on the outer membrane (Bertozzi Silva et al., 2016). *P. aeruginosa* phage can also use surface-exposed outer membrane porin (OprM) of the MexAB and MexXY efflux pump proteins as a receptor (Chan et al., 2016). Bacteria can develop phage resistance by either eliminating or modifying these receptors. This intense survival pressure has led to the regulation of efflux pumps by compromising their function (Gurney et al., 2020). Some phages can counter this evolutionary upgrade by either adapting to the new receptor or altering their receptors (Samson et al., 2013; Habusha et al., 2019). This study demonstrates a coevolution-based strategy that opens a new window to treat MDR *P. aeruginosa* with a phage that can induce genomic mutations, SNPs, or deletions in its bacterial hosts, producing a compromised drug-resistance system. This phage is referred to as PIAS (Phage Induced Antibiotic Sensitivity). The phage infection resulted in a sequential emergence of two mutants: green/pale mutants, followed by brown mutants. A coevolutionary process also led to tail-fiber protein mutants of the PIAS that could infect the green/pale mutant host. The PIAS uses the MexXY-OprM efflux pump as the receptor. This was confirmed with transposon knock-out mutants derived from *P. aeruginosa* strain PA01 (Jacobs et al., 2003). Mutants selected after multiple rounds of infection with PIAS displayed phage resistance by compromising antibiotic resistance. We propose a combined PIAS/antibiotic treatment to combat MDR *P. aeruginosa* infections.

## Materials and Methods

### Bacterial Strains, Growth Conditions, and Antibiotic Sensitivity

Bacterial isolates were acquired from the Clinical Microbiology Laboratory at the University of Szeged over the course of a year. The strains were identified by MALDI-TOF MS and 16S ribosomal RNA gene sequencing using universal 16S rDNA bacterial primers: 27F (AGAGTTTGATCATGGCTCA) and 1492R (TACGGTTACCTTGTTACGACTT). All bacterial strains were grown aerobically at 37°C in either Luria Broth (LB) or Agar (LA). We screened all the strains for serotype according to the BioRad 16 monovalent sera kit numbered from 1 to 16 by the classification established by the International *Pseudomonas* Sub-Committee. Primary antibiotic resistances of the wild-type and mutant strains were determined by disk diffusion antibiotic susceptibility tests (BioRad). 300 μL of overnight (OVN) grown bacterial culture was spread onto an LA plate and allowed to dry for 10 min, then antibiotic-containing disks were applied to the agar. Plates were incubated at 37°C for 12 h and sensitivity was estimated on the basis of the size of the inhibition zone. Proper antibiotics (gentamicin, fosfomycin, ceftazidime, and tetracycline) were chosen and broth microdilution method was used the establish for minimal inhibitory concentrations (MIC). Four antibiotic stocks were diluted to different test concentrations in LB medium. The OVN grown culture was diluted to ∼2 × 10^5^ cfu/mL in LB. MICs for the above antibiotics were determined by dispensing 100 μL of a given antibiotic concentration and 100 μL of diluted bacteria into the wells of a 96-well plate and incubating at 37°C with shaking for 12 h. Bacterial growth was monitored by a spectrophotometer (Thermo Multiskan Ascent Plate Reader) at 600 nm. The lowest antibiotic concentration, at which no change in OD could be observed, was considered as MIC. Each strain was tested in triplicate.

### Bacteriophage Isolation and Stock Preparation

We assayed different clinical sources, including swabs from an infected wound and blood samples, for *P. aeruginosa*–specific bacteriophages. We homogenized solid and liquid samples in 5 ml of phage suspension buffer (10 mM Tris–HCl, pH 7.6, NaCl 0.4%, gelatin 0.1%), centrifuged the homogenate, and filtered it through 0.22 μm pore-size filters (Sigma). The filtrate was added to 25 ml LB broth containing 1 ml overnight inoculant of various *P. aeruginosa* MDR strains and incubated it at 37°C for 24 h. We added chloroform (0.1%, final concentration) to the culture then incubated it at 37°C for a further 5 min. After removing bacterial cell debris by 10 min centrifugation at 9,000 *g* at 4°C, we filtered the suspension through a 0.22 μm pore-size filter. We determined the presence of phages in the filtrates by spot assay, as described previously (Mazzocco et al., 2009).

### Phage Purification and TEM Electron Microscopy

Amplified high-titer phage stocks were obtained through liquid broth amplification (LB medium). We adapted the protocol from Cui et al. (2017), with minor modification. Single PIAS plaques from a plate containing PIAS pregrown on the individual host inoculum (1 ml for 10 ml fresh broth) were incubated at 37°C for 12 h. We prepared the phages from continuous consecutive passages: each passage was centrifuged, filtered, and combined with fresh inoculum every 12 h and incubated for 48 h to minimize the risk of host coevolution. We treated the lysed liquid culture obtained after incubation with chloroform (final concentration of 0.2%) for 30 min with gentle shaking to kill the remaining bacteria, and we then added NaCl (0.5 M) and incubated the culture for 1 h at 4°C. We removed bacterial debris by 10 min centrifugation at 8,000 × *g* and 4°C. We coagulated the phages in the supernatant overnight using polyethylene glycol PEG8000 (100 g/L) at 4°C. After 30 min centrifugation at 12,000 × *g* and 4°C, the collected pellet was dissolved in phage suspension buffer (10 mM Tris–HCl, pH = 7.6, NaCl 0.4%, gelatin 0.1%, 1 mL phage suspension buffer per 100 mL original volume) and vortexed. We added an equal volume of chloroform to remove PEG8000 and other debris and carried out 15 min centrifugation at 5,000 × *g* and 4°C. The aquatic phase (upper phase) with a high titer of phages was collected and stored at 4°C. We viewed samples of the purified phage under a JEM-1400 Flash transmission electron microscope (JEOL; Tokyo, Japan) at the Biological Research Center, Szeged, Hungary. We deposited 5 μl of the sample onto formvar-coated 150 mesh copper grids (Electron Microscopy Sciences; Hatfield, PA, United States). We used the edge of a filter paper to remove the excess fluid after 30 s, and then samples were contrasted by adding 20 μl 1% uranyl acetate (Electron Microscopy Sciences) in 50% ethanol (Molar; Halasztelek, Hungary) for 3 min (three times). After removing the superfluous staining solution, samples were dried in a petri dish for 2 h before taking the electron microscopic picture. We screened samples systematically at 10,000× magnification to localize the presence of phages on the grid. We then recorded healthy, unbroken phages at 80,000–100,000 × magnification with a 16 MP Matataki Flash sCMOS camera (JEOL).

### Phage Genome Sequencing and Phylogenetics

Phage DNA isolation was carried out using the High Pure Viral Nucleic Acid Large Volume Kit (Roche Cat No. 05114403001). We sequenced the phage genomes on Illumina MiSeq platform and achieved coverages of at least 50x. The sequences were filtered by BBduk^1^ and assembled using Spades (v.3.13.1) with default settings and the “careful” option (Nurk et al., 2013). From the output, we used the “contigs.fasta” files for annotation with Prokka (v.1.14.6) (Seemann, 2014). We downloaded all other *Pseudomonas* phage gene sequences from Uniprot and used them as trusted sequences for annotation. Phage phylogeny was carried out using VICTOR (Meier-Kolthoff and Göker, 2017), and comparison was performed by Brig (Alikhan et al., 2011). The phage sequences were uploaded to the NCBI database (Bioproject: PRJNA720536).

### Determination of PIAS Phage Infection Efficiency

The PIAS phage infection efficiency was assayed in 96-well plates for 24 h. All samples were run in triplicate in three different experiments. We added 500 μl of overnight test strain culture to 10 ml of LB in a 250 mL flask and incubated it at 37°C, shaking it at 200 rpm, until an OD_600 nm_ ≈ 0.6 (∼1 × 10^8^ CFU/ml) was reached. Once the required culture density was obtained, we put 90 μL of bacteria into the wells. We treated bacterial samples with 10 μL of phage stock with different multiplicity of infection (MOI) (0.1, 1.0, and 10.0). The control was mock-treated with sterile phage buffer. We measured the OD_600 nm_ every 3 h for 24 h using a Thermo Multiskan Ascent Plate Reader. The latent period and burst size were determined according to the protocol of the Jeffrey Barrick laboratory (Heineman and Bull, 2007). To determine the time required for PIAS adsorption to the wild-type and transposon mutants, adsorption assay was performed following the protocol of Kropinski (2009). We determined the mutant frequency *via* the previously described method of Shen et al. (2018).

### Bacterial Genome Sequencing and Genome Comparison

Bacterial genomic DNA was extracted from mutant and parental strains using GenElute Bacterial Genomic DNA kits (SIGMA, NA2110_KT). We sequenced 5 wild-type strains and 15 mutants. We first prepared libraries using the Nextra XT kit (Illumina) and sequenced them by a MiSeq sequencer (Illumina Inc.). Paired-end sequencing with 150 bp read length was used to generate reads. Quality assessment was carried out with FastQ (Wingett and Andrews, 2018). We trimmed raw reads to remove adapter sequences and PhiX174 contamination using BBduk. Sequence reads were assembled using Unicycler (version v0.4.7) (Wick et al., 2017). The assembled sequences were annotated with Prokka (version 1.14.6) (Seemann, 2014) using all *P. aeruginosa* proteins. We downloaded 6,661,531 protein sequences belonging to *P. aeruginosa* from Uniprot and used these as trusted proteins for annotating assembled contigs in Prokka. BBMap utilities were applied to obtain assembly metrics’ statistics. To identify genome deletions, all reads were mapped back to the reference *P aeruginosa* PAO1 genome. Mapped reads were analyzed using Samtools (Li et al., 2009) and visualized using an Integrated Genome Viewer (Robinson et al., 2011). The annotated genomes were compared to identify the missing genes across the strain and mutants using Roary (Page et al., 2015). We identified SNPs in the green mutants by mapping the reads to their respective reference strain using Breseq (version: 0.35.4) (Deatherage and Barrick, 2014).

### Membrane Integrity Measurements

Membrane sensitivity can be measured using the membrane-damaging agent bile acid (Lázár et al., 2018). We studied the membrane integrity of the mutant strains by measuring their sensitivity to bile acid. The experiments were carried out in 96 well plates for 24 h. We added 10 μL of overnight test strain culture to 1 mL of LB supplemented with 1–7% bile acid. We plated 200 μL from each concentration. The mock-treated control contained sterile buffer without bile acid. We measured the OD_600 nm_ over 24 h (Thermo Multiskan Ascent Plate Reader). Data are reported in graphics, with each point representing the average ± standard deviation of three wells from different experiments.

### Screening the Knock-Out Mutants for PIAS Phage Sensitivity

Based on the genome sequences of the green and brown mutants, we purchased 12 transposon knock-out mutants from the Transposon Mutant Collection of the University of Washington (Genome Sciences, Manoil Lab) (Supplementary Table 3). For the drop assay, we added 200 μL of each mutant OVN culture to 5 mL of molten LB soft agar (0.6% agar) and overlaid the mixture onto a 1.5% LB agar plate to generate a bacterial lawn. After solidification, we spotted 5 μL volumes of serially diluted (10-fold dilution) PIAS phage suspensions and then incubated the plates at 37°C. We determined the PIAS susceptibility of tested bacteria based on the formation of a single clear plaque or a bacterial growth inhibition zone on the plates.

### Isolation of Co-evolved Phages

We used the previously published double agar plaque assay protocol with slight modification (Kropinski et al., 2009). The Host strains were grown at 37°C with agitation until reaching ∼2 × 10^8^CFU/ml. We then mixed 200 μl cultures with the same number of PIAS (MOI 1.0) in the presence of 5 mM of CaCl_2_. After 20 min incubation at room temperature, the suspension was mixed with 5 mL warm, soft agar (4 g/L) and poured onto on LB agar. Following 64 h incubation at 37°C, the plate’s soft agar was scraped and homogenized in 5 mL of phage suspension buffer. This suspension was centrifuged and filtered through a 0.22 μm pore-size filter (Sigma). Finally, we diluted the phage suspension in 10-fold increments and plated it with overnight bacterial mutant culture (SNP mutant). After 24 h, we isolated single plaques and carried out purification and genome sequencing as described above.

### Elimination of Bacteria With Combined PIAS Phage and Antibiotic Therapy

Following a spot test, we mixed 100 μl from an overnight-grown sensitive strain (2 × 10^8^ ± 0.2 × 10^8^ cfu/mL) with an equal number of PIAS phage (MOI 1.0) and 3 mL of the molten soft agar (0.5%) and overlaid it on the surface of the solidified LB agar (1.5%). We allowed each overlay to solidify for 20 min and incubated them at 37°C for 48 h. To investigate the combination therapy, we plated the above mixture on solid antibiotic agar with soft agar containing various antibiotics [e.g., fosfomycin (FSF) 30–100 μg/mL]. In addition, we spread culture without phage as control onto the antibiotic-containing agar plates. The planktonic assay was carried out in 96 well plates. We run all samples in triplicate with three independent experiments. We added 500 μl of overnight culture to 10 mL of LB in a 250 mL flask and incubated it at 37°C, shaking it at 200 rpm, until reaching OD_600 nm_ ≈ 0.6 (∼1 × 10^8^ cfu/ml). After the required culture density was attained, we placed 180 μL of it into the wells. Bacterial samples were mixed with 10 μL of phage (MOI 1.0) and 10 μL antibiotic stock solutions in various concentrations, ranging from 10 to 100 μg/mL (two controls were also prepared for testing the phage and antibiotic sensitivities, separately). We measured the OD_600 nm_ every 3 h for 24 h using a Thermo Multiskan Ascent Plate Reader. We plotted the least inhibited antibiotic concentration, with each point representing the mean ± standard deviation of three wells from the different experiments.

### *In vivo* Rescue Experiments in a Mouse Lung Infection Model

*Pseudomonas aeruginosa* clinical strain PA16 was used as the host bacterium. We used PIAS phage and FSF as therapeutic agents. Table 3 shows the basic experimental setup. We assayed the potential therapies in a lung infection model using 8–10-week-old BL67 black female mice (C57/BL67 weight: 18–25 g, origin: Charles River Laboratories). The animals were maintained in room with standard temperature (23°C ± 1°C) using 12-h dark/12-h light cycles for the bacterial challenge. PA16 grew up to OD_600 nm_ ≈ 0.5 in 50 mL of LB broth at 37°C with shaking at 120 rpm. The bacterial suspension in the log phase was centrifuged (for 1 min at 12,000 rpm), washed with PBS, and the OD_600 nm_ was set to 1.0. The suspension was diluted to 5× and 15× diluted suspension was administered through the nostrils of intraperitoneally anesthetized mice (see Table 3). The anesthetic used was one dose of 70 μl Combo solution [10 ml PBS, 10 ml Calypso (50 mg/ml injection; Richter Gedeon)] and 150 μl Primazin (Alfasan-Woerden, Netherlands). Five days after the bacterial challenge, we administered PBS, FSF, PIAS phage, and their combinations for both the control and the treatment groups once intraperitoneally. All mouse experiments were conducted according to the guidelines of the European Federation for Laboratory Animal Science Associations. The Animal Welfare Committee approved all protocols and procedures involving animals of the Enviroinvest Co. (Permit Number: BAI/35/867-6/2019). To investigate whether any phage-resistant mutant colonies were formed during the *in vivo* phage treatment, we removed the organs after the end of the post-infection treatments. The lung, spleen, and brain were aseptically homogenized in sterile PBS using a tissue homogenizer. We plated homogenized tissue aliquots onto a *P. aeruginosa*–selective nutrient agar and incubated them at 37°C for 48 h. We selected 20 random colonies and screened them against PIAS, FSF, and E-PIASs sensitivities. In addition, we screened homogenized aliquots for phages using the double-layer agar method with wild-type PA16 bacteria.

### Statistical Analysis

Statistical analysis was performed using one-way analysis variance (ANOVA) with dunnett’s multiple comparisons test for the line chart and two-way (ANOVA) was performed with Tukey’s multiple comparison test for bar graph in GraphPad Prism Version 8.4.2 (GraphPad Software). Differences were considered statistically significant with a *P*-value if *P* < 0.001 ***, if *P* < 0.01 **, if *P* < 0.05 *. *ns* = not significant.

## Results

### Experimental Model and Subject Details

#### Bacterial Hosts

We collected strains from diverse clinical samples and typed them using serotyping and antibiograms. We isolated and identified 25 strains that were all resistant to at least one antibiotic class. We chose five MDR strains (PA16, PA17, PA21, PA22, and PA59) for our study. The antibiotic sensitivities of these strains were screened by the disk diffusion method, followed by a minimum inhibitory concentration (MIC) assay. In the case of four strains, the MIC of gentamicin (aminoglycosides) was ≥45 μg/ml; for two strains, the MIC of ceftazidime (antipseudomonal cephalosporins) was ≥45 μg/ml. For all five strains, the MICs of fosfomycin (phosphonic acid) and tetracycline (polyketide) were ≥180 and ≥93 μg/ml, respectively. We extracted and sequenced genomic DNA from each bacterial isolate. The assembled data generated multiple contigs across all bacterial strains. As each strain had multiple contigs, we used PAO1 as the reference genome and mapped all the reads to this reference (Bioproject: PRJNA57945). The genome sizes ranged from 6.3 to 6.9 Mbp.

### Phage Isolation and Characterization

**FIGURE 1:**
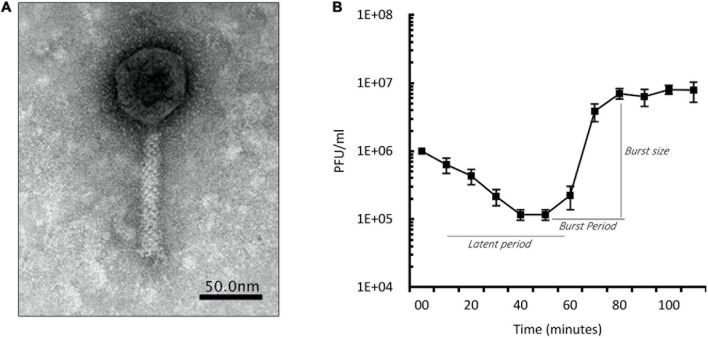
Phenotypic characterization of PIAS. **(A)** Transmission electron microscopy image of PIAS shows a contractile tail length of 110 nm. The head of PIAS has a length of 55 nm. The scale bar shown is ∼50 nm. **(B)** One-step growth curve of PIAS; plot represents the values of three independent experiments. The latent phase of PIAS takes approximately 55–60 min, and the phage can produce approximately 110 phage progeny per infected cell.

We isolated 11 phages and chose PIAS and PAPSZ1 (*P. aeruginosa* Phage SZEGED 1) for further studies based on their ability to induce phenotype changes in the target bacterium. We characterized PIAS phage by electron microscopy (Figure 1A), host specificity, one-step growth experiments, and genome sequencing. PIAS is a 92.3-kb *Myoviridae* phage that produced massive plaques on all sensitive clinical strains surrounding halos. It infected eight out of 25 clinical strains. No host serotype specificity of the phage could be observed. Using the PA16 strain, we found the latent period of PIAS to be between 55 and 60 min and the burst size to be approximately 110 pfu per infected cell (Figure 1B). As shown in the graph, approximately 90% of the phages were adsorbed to host cells in 30 min.

### Phage Genome Sequencing and Comparison

**FIGURE 2:**
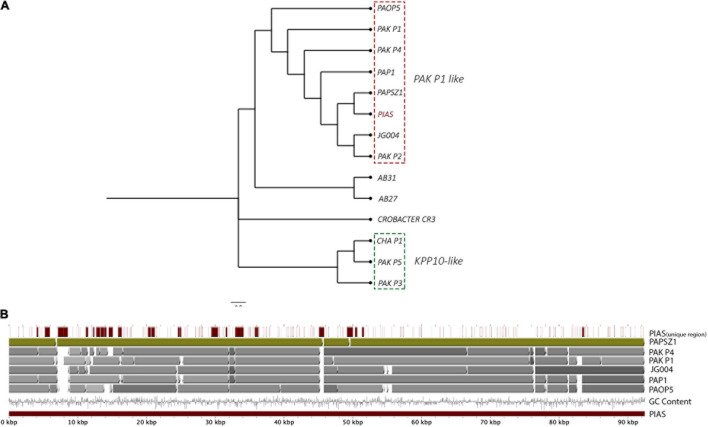
Genome-wide comparison of PIAS and PAPSZ1 to other selected *P. aeruginosa* phages. **(A)** Phylogenetic analysis of PIAS to determine closely related phages. **(B)** Whole genome comparison of PIAS to closely related phages.

The complete genome of PIAS was sequenced, assembled (92.3 kb) and annotated (Bioproject: PRJNA722489). Phage annotation against phage sequences in Uniprot provided hits for only 15 out of 181 predicted CDS regions (see Bioproject). Phage phylogeny revealed that PIAS was closely related to the phages PaP1 (Lu et al., 2013) and PaoP5 (Shen et al., 2016; Figure 2A; Supplementary Table 1). Previous studies have identified these phages as a separate group called PAK-P1-like phages (Henry et al., 2015). This group of phages clusters separately from other *P. aeruginosa* phages. Multiple whole genome comparisons of PIAS against other Pak-P1 phages show that the phages have shared syntenies (Figure 2B).

### Formation of PIAS Phage-Resistant Strains

**FIGURE 3:**
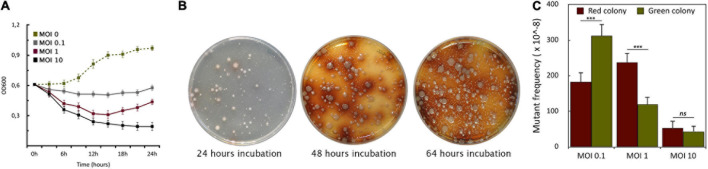
Formation of phage-induced PA16 mutants. **(A)** The graph shows the growth curves of PA16 host infected with PIAS at different MOIs (0.1, 1.0, and 10). Error bars represent SEM for three replicates. **(B)** The plates show the appearance of PIAS resistant colonies after PIAS infection. The color of mutant colonies shifted from green or pale to brown over a period. **(C)** MOI dependence of the frequencies of the green and brown colony formation.

To assess the host killing efficiency of PIAS, we grew a PA16 culture in Luria-Bertani (LB) broth and infected it with PIAS, using different MOI (Multiplicity of Infection) values (0.1, 1.0, and 10.0). The bacterial growth was monitored by measuring optical densities at OD_600_. In each case, the phage infection inhibited bacterial growth, which was progressively more prominent with increasing MOI. The optical density of the culture decreased at approximately 3 h post-infection with all three MOI values (Figure 3A). Bacterial growth ceased at a high phage titer (MOI 10). When we used an equal ratio of phage to bacteria (MOI 1), bacterial growth increased slightly after 12 h. At a lower phage titer (MOI 0.1), bacterial growth was higher than observed when the cells were infected at both MOI 1 and MOI 10, but it was still lower than in the case of non-infected cells.

Phage infection of PA16 led to the sequential appearance of colonies with two phenotypes (Figure 3B). The initial set of colonies that arose was green/pale, and this was followed by a set of brown colonies. Colonies of these two phenotypes could be observed with the other four strains. However, the time elapsed before the appearance of the differently colored variants depended on strains and MOI values. In a double-layer soft agar plate, the mostly green or pale colonies appeared after 16–48 h incubation. Notably, no green or brown colonies appeared in the absence of phage infection after several replating of the hosts, therefore the phenotypic (and genotyping see below) changes were evidently phage-induced or phage-provoked. At higher MOI, there were fewer phage-resistant colonies across many clinical strains (Figure 3C). The average frequencies of the five studied strains were: at MOI 0.1: ∼311 ± 20 × 10^–8^ for green and ∼183 ± 25 × 10^–8^ for brown colonies; at MOI 1: ∼120 ± 22 × 10^–8^ for green and ∼238 ± 24 × 10^–8^ for brown; at MOI 10: ∼43 ± 10 × 10^–8^ for green and ∼53 ± 10 × 10^–8^ for brown colonies. A higher MOI always generated a lower incidence of phage-resistant colonies. Therefore, we used MOI 1 as a standard for further experiments.

### Genomic Characterization of Phage-Resistant Strains

**FIGURE 4:**
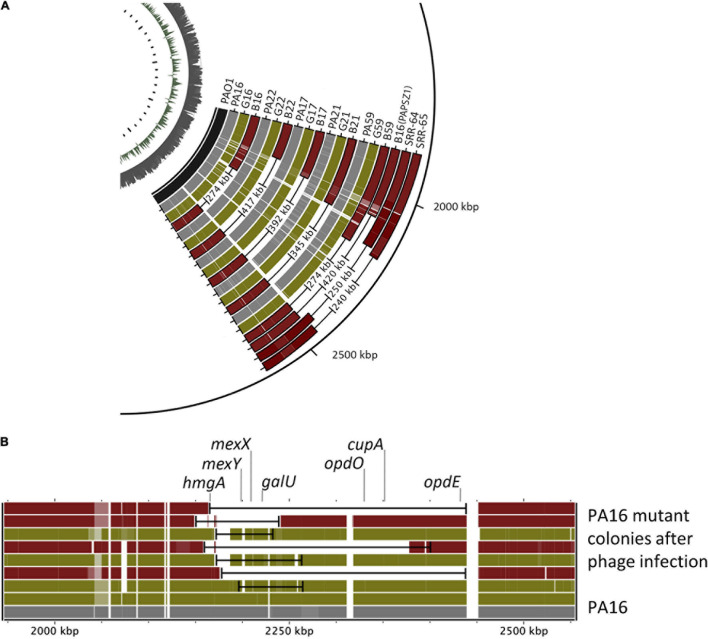
Comparison of the genomic regions of the wild-type and various phage-provoked *P. aeruginosa* mutants. **(A)** The genomic regions of the wild type, green (G) and brown (R) PA16, PA17, PA21, PA22, PA59 mutants induced by either PIAS or PAPSZ1. PAO1 is the reference genome, and the genomic deletions published in (40) (SRR-64, SRR65) are also shown for comparison. **(B)** Comparison of genomic deletions in various green and brown PA16 mutants in the region of 2000–2500 kbp.

We sub-cultured single mutant colonies of both mutant types and determined their genome sequences. Again, we used the PA01 genome as the reference genome. As shown in Figures 4A,B, the genomes of all green colonies harbored SNPs and relatively small deletions (20–80 kbp), whereas we observed large deletion (274–417 kb) in all the brown colonies. Most importantly, the mutated genomic regions of the green and the brown mutants were the same. Deeper analysis of the mutations in the green colonies showed that the common SNPs occurred in the *mexY* gene (Table 1), which might hinder the phage infection of the green mutant. The more significant deletion in the brown mutants, occurring in the genome’s 2000–2500 kb region, removed the *mexY* gene, with many genes involved in membrane integrity, transport, *quorum sensing*, pigmentation, and antibiotic resistance (Supplementary Table 2). It is vital to consider that the *oprM* gene does not lie in the deleted region; instead, it is positioned between 475 and 480 kb. We compared the mutated genomic regions of the brown mutants to those of a previous study by Shen et al. (2018) (Figure 4A, SRR64, SRR65). Despite using different phages and hosts, the deleted regions in the brown mutants were almost similar in both studies. We note that Shen et al. used the PaoP5 phage, which falls in the PAK-P1-like phage family (Figure 2A). The pattern of smaller deletions in various PA16 green mutants (Figure 4B) suggested that the formation of the more significant deletion might occur *via* a continuous multistep evolutionary process that began with the emergence of SNPs, followed by smaller and more significant deletions.

**TABLE 1:** Single nucleotide genetic variations in the *mexY* gene of the green/pale mutant of various *P. aeruginosa* strains.

Mutated gene	Strain	Mutation	Annotation
*MexY*-Multidrug efflux RND transporter subunit (OrpM-MexXY)	G16	C → A	T408N (ACC → AAC)
	G21	829 bp	MC
	G22	G → A	Intergenic (−/+520)
	G17	G → T	E658 (GAG → TAG)
	G59	80 bp	MC
	IVA16	C → A	T408N (ACC → AAC)
	IVB16	C → A	T408N (ACC → AAC)
	IVC16	C → A	T408N (ACC → AAC)
	IVD16	C → A	T408N (ACC → AAC)

*All strains are resistant against PIAS but can be infected by E-PIASs. The MIC values were also reduced for these mutants.*

### Membrane Integrity

A break in the integrity of the cell membrane immediately compromises its essential role as a barrier and can endanger the affected cell. Whole-genome analysis of the mutant bacteria revealed that they had lost many membrane-related genes (Supplementary Table 2). To investigate the impact of these gene deletions, we measured the membrane sensitivity to bile acid. All five brown mutants exhibited higher sensitivity to bile acid than did wild-type bacteria (Supplementary Figure 1). The average MIC concentrations of bile acid for wild type, green and brown mutants were 6% (±0.5%), 4% (±1%), and 2.5% (±0.5%), respectively. Conversely, the bile acid sensitivity of the green mutants was almost similar to that of wild type.

### Screening Receptor-Based Knock-Out Library for Phage Sensitivity and Adsorption

**FIGURE 5:**
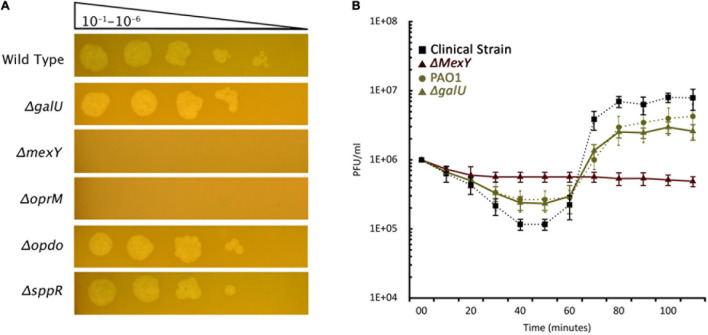
PIAS phage sensitivity of PAO1 transposon mutant strains. **(A)** PIAS plaque spot assay on various PAO1 transposon mutants (Supplementary Table 3). **(B)** PIAS adsorption assay with the PA16 and PAO1 wild-type strains as well as selected PAO1 transposon mutants. Each experiment was done in triplicate. Error bars represent SEM for three replicates.

Bacteria evolve phage resistance by alerting, losing, or masking phage receptors from binding. In such a mutant, the phage can no longer be adsorbed and multiply. The genomic analysis of the mutants identified few potential targets as phage receptors. We screened 12 knock-out mutants (Supplementary Table 3) for PIAS sensitivity. The Δ*oprM* and Δ*mexY* mutants displayed complete resistance to PIAS, but the Δ*galU* strain (GalU is responsible for the biosynthesis of LPS core region) was still sensitive (Figures 5A,B). We then quantified unadsorbed phages after a brief incubation period, revealing defects in the binding. Adsorption was lower in both the wild-type PAO1 strains and transposon knock-out mutants than in our wild-type clinical strains. Adsorption at a set point compared to the wild type of clinical strain was ∼40% less in PAO1 and Δ*galU* PAO1 strains, and we observed no adsorption with Δ*mexY* and Δ*oprM* PAO1 mutants (Figure 5B).

### Isolation of Co-evolved PIAS Phage Mutants, Evolved PIASs

To study the (evolutionary) reason of the two types of mutants, we first isolated early pale mutants of PA16, PA22, PA21, and PA17. We used whole-genome sequencing to confirm the SNPs. We were then able to isolate phages capable of infecting these green mutants from a previously infected plate. Phage lysates were collected and plated with these green mutants. We purified, propagated, and sequenced (Bioproject: PRJNA722489) single plaque capable of infecting green mutants from various hosts. Comparison of their genomes to that of the wild-type PIAS revealed that phages had SNPs in the phage tail fiber protein (Table 2). This mutation enabled the phages to infect the green bacterial mutants.

**TABLE 2:** Single nucleotide genetic variations in the phage tail fiber protein of various E-PIAS phages isolated on various hosts.

Mutated gene	E-PIAS	Mutation	Annotation
Tail fiber protein	E-PIAS H16	T → G	I639S (ATC → AGC)
	E-PIAS H22	T → G	I639S (ATC → AGC)
	E-PIAS H21	G → A	H635Y (TGA → TAA)
	E-PIAS H17	C → T	R491H (GCG → GTG)
		G → T	T291K (CGT → CTT)

### Challenging Bacteria With Phage and Phage–Antibiotic Combinational Therapy

**FIGURE 6:**
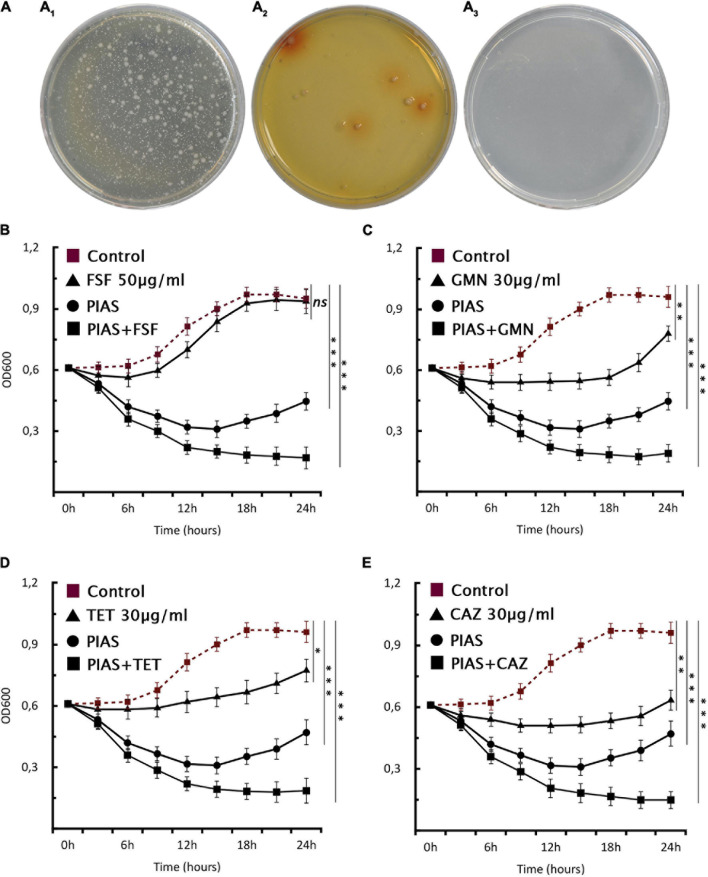
Phage–antibiotic combinational therapy *in vitro*. **(A)** Combination therapy on solid agar, *P. aeruginosa* PA16 plated **(A_1_)** with antibiotic (30 μg/ml GMN), **(A_2_)** with PIAS (∼2 × 10^8^ pfu), and **(A_3_)** with PIAS (∼2 × 10^8^pfu) and antibiotic (30 μg/ml GMN). **(B–E)** The graphs show the single and combined effects of phages and antibiotics on cell growth as compared to the non-treated cells. The PIAS (∼2 × 10^8^pfu) and the following antibiotics: **(A)** FSF 50 μg/ml, **(B)** GMN 30 μg/ml, **(C)** TET 30 μg/ml, and **(D)** CAZ 30 μg/ml were used for the combinational therapy.

One of the most discussed potential drawbacks of phage therapy is the possible rise of phage-resistant bacterial mutants that evolve and proliferate during treatment. Genome analysis of the evolved bacterial mutants revealed that the bacteria had an altered membrane-related gene product MexY (of MexXY-OprM system) for self-defense against PIAS. This protein plays a key role in the RND drug efflux system that is involved in antibiotic resistance (Masuda et al., 2000). We first determined the MIC values of all five clinical strains and their mutants for four antibiotics (Table 3). In all cases, the phage-induced mutants were substantially more sensitive to all antibiotics. This means that the formation of phage resistance comes at the cost of increased drug sensitivity. We used this window as a strategy for the comprehensive eradication of mutants by treating the PA16 wild-type strain with an antibiotic (fosfomycin, gentamycin, tetracycline, and ceftazidime) combined with PIAS. A control plate with gentamycin but without phage had visible colonies after 24 h (Figure 6A_1_), and the mutant formation described above could also be observed (Figure 6A_2_) after phage infection. The PIAS/antibiotic combination prevented the formation and growth of mutants after 48 h incubation (Figure 6A_3_). We confirmed this *via* a planktonic assay using four antibiotics (Figures 6B–E). We found that combination therapy outperforms either phage or antibiotic alone. Notably, the same strategy can be achieved by many other antibiotics to which bacterial resistance is based on the RND system.

**TABLE 3:** Drug sensitivity of the wild-type and phage-provoked green and brown mutants of various MDR *P. aeruginosa* clinical isolates.

Antibiotic	Strain	Antibiotic MIC		
		Wild-type	Green mutant	Brown mutant
Tetracycline	PA16	173.3 ± 11.1	19.3 ± 1.5	16.6 ± 1.5
	PA21	126.6 ± 11.5	16.6 ± 1.5	12.6 ± 1.5
	PA22	93.3 ± 11.5	18.6 ± 1.5	14.6 ± 1.5
	PA17	126.7 ± 11.5	21.3 ± 1.5	18.6 ± 1.5
	PA59	130.0 ± 10.0	18.7 ± 1.5	18.7 ± 1.5
fosfomycin	PA16	201.6 ± 2.8	29.3 ± 3.0	20.3 ± 2.5
	PA21	180.0 ± 0.0	28.0 ± 0.0	21.3 ± 4.1
	PA22	200.3 ± 20.0	31.6 ± 2.8	27.6 ± 2.5
	PA17	193.3 ± 11.5	26.6 ± 1.5	31.6 ± 2.8
	PA59	193.3 ± 11.5	40.0 ± 5.0	35.6 ± 5.0
Ceftazidime	PA16	30.0 ± 2.0	8.6 ± 1.5	5.3 ± 1.1
	PA21	45.3 ± 1.1	7.3 ± 1.5	6.6 ± 1.2
	PA22	30.6 ± 1.1	9.3 ± 1.2	5.3 ± 1.2
	PA17	46.7 ± 1.1	10.0 ± 2.0	6.6 ± 1.1
	PA59	46.7 ± 1.1	17.6 ± 2.5	11.6 ± 2.5
Gentamycin	PA16	51.6 ± 2.8	21.6 ± 2.5	15.0 ± 2.5
	PA21	61.7 ± 2.8	21.6 ± 2.5	15.0 ± 2.5
	PA22	51.6 ± 2.8	12.0 ± 2.5	10.0 ± 2.5
	PA17	45.0 ± 5.0	12.0 ± 2.5	10.0 ± 2.5

### *In vivo* Rescue Experiment in the Mouse Lung Infection Model

Table 4 summarizes our experimental setup and results. We first tested the effects of controls - phosphate buffered saline (PBS), high dose of phage, and FSF - on the mice. None of these had a drastic effect on the animal during the 10-day monitoring period (Table 4, Group 1–3). Infection doses in the applied bacterial lung model adapted to PA16 were established in preliminary studies. Administration of 5 × 10^6^ cfu/mouse was fatal and killed all experimental animals in 2 days. Conversely, administration of one-third of this bacterial load [15× dilution (15×)] ensured the survival of 75% of the animals (Table 4, Group 4). Therefore, the experiments with 5 × 10^6^ cfu/mouse doses will be discussed here. In the case of the 5× bacterial dilution, the survival ratios were modified only minimally if either PIAS - at any applied MOI (0.2, 1, and 5) - or 1 mg FSF was administered intraperitoneally to the challenged groups. However, this situation changed drastically if 1 mg FSF and PIAS were used in combination as therapeutic agents. In the case of PIAS (MOIs 1 and 2) combined with FSF, 25% of the animals survived, and their lifespans were elongated in some cases. In the case of MOI 5 applied with FSF, 75% of the animals survived (Table 4, Group 7). Combination therapy ensured 100% survival of all mice in groups in which the bacterial challenge was performed with the 15× diluted bacterial inoculum.

**TABLE 4:** Treatments and survival rates in the *in vivo* rescue experiments in the mouse lung infection model.

Group	Bacterium dilution	Bacterium cfu/mouse	FSF mg/mouse	Phage MOI	Phage pfu/mouse	Survival 10 days
**1**	**A. PBS control**					
	-	-	-	-	-	100%
**2**	**B. PIAS control**					
	-	-	-	-	25,000,000	100%
**3**	**C. fosfomycin control**					
	-	-	1.0	-	-	100%
**4**	**D. Bacterium PA16 dilution controls**					
	5x	5,000,000	-	-	-	0%
	15x	1,600,000	-	-	-	75%
**5**	**E. Bacterium PA16 + Phage PIAS_PA16g_**					
	5x	5,000,000	-	0.2	1,000,000	0%
	5x	5,000,000	-	1.0	5,000,000	0%
	5x	5,000,000	-	5.0	25,000,000	25%
**6**	**F. Bacterium PA16 + fosfomycin**					
	5x	5,000,000	1.0	-	-	0%
**7**	**G. Bacterium PA16 + fosfomycin + Phage PIAS_PA16g_**					
	5x	5,000,000	1.0	0,2	1,000,000	25%
	5x	5,000,000	1.0	1.0	5,000,000	25%
	5x	5,000,000	1.0	5.0	25,000,000	75%

We recovered PA16 bacterial colonies from all organs, including the brain, spleen, and lung. We screened 20 isolates from each organ against FSF, phage PIAS, and E-PIASs. We recovered fewer bacteria from the brain than from either spleen or lung. Conversely, we found a higher number of bacterial mutants in lung than in other organs. Approximately 4% of the isolated colonies from lung and 2% of isolates from spleen were mutants. We isolated one brown colony and two green colonies from the lung. The brown colony was sensitive to FSF (>50 μg/ml), and the two green colonies displayed decreased MIC for FSF and were sensitive to E-PIAS. Two mutant colonies in spleen had decreased MIC for FSF and were sensitive to E-PIAS. We found no mutant colonies in brain isolates. We also screened the homogenized lung solution for phages. We selected multiple plaques and screened them against PA16g mutants and wild-type strains. Phage isolates were able to infect the wild-type PA16 but not the PA16g mutant strains. We could not detect any phages in the other organs.

## Discussion

Bacteriophages are the only antimicrobial entities that show precision in choosing a host and self-replicate by continuously co-evolving with their host. The idea of using phages to combat bacteria is not new. The phagoBurn study of burn patients infected with *Escherichia coli* and *P. aeruginosa* are some recent examples of such application (Jault et al., 2019). However, the idea of using phage therapy to its full potential by utilizing an evolutionary window is unique. Many interactions occur during the phage infection cycle. These interactions happen in different stages, beginning with the early reversible binding of the virion and continuing till the end of the viral release (Thomassen et al., 2003).

In many cases, the *O*-antigene of the LPS layer serves as a receptor for phage adsorption in *P. aeruginosa* (Bertozzi Silva et al., 2016). One of the most striking features of *P. aeruginosa* is its remarkable capacity to develop fast resistance *via* chromosomal mutations; this helps it combat antimicrobial treatments and/or phage infection. An SNP in the phage receptor is sufficient to stop phage infection in *P. aeruginosa* and *Klebsiella pneumonia* (Lim et al., 2016; Li et al., 2018; Hesse et al., 2020). The phage genome can also be evolved to counteract the bacterial defense mechanisms by inducing mutation in its genome, as it was described for *Bacillus subtilis* (Habusha et al., 2019).

The PIAS described in this study could inhibit and eradicate many tested clinical strains at high MOI values. At low MOIs, hosts undergo multistep evolution by generating changes in their genomes to block phage infection. This is a critical issue to review, as the uneven distribution of the bacteria and phages in real applications will result in different MOI values in different organs. The first line of bacterial defense caused the growth of green colonies containing SNPs and small genomic deletions. These mutations appeared shortly after single plating. A comparative study of multiple green mutants from a single clinical strain revealed small deletions with some variations (Figure 4B). Nevertheless, the patterns of the more minor genomic deletions in the green mutants suggest a multistep bacterial evolution process, which begins with SNPs followed by smaller to more significant deletions that interfere with phage infection. Apart from observing random and more minor deletions in the 2000–2500 kb region, we were unable to isolate a mutant with single-gene deletion responsible for phage resistance. All these green mutants had either SNPs in the *mexY* gene and/or smaller deletions. Our experiments clearly showed that these mutations defended the bacterial lineages from phage infection. This was counteracted by PIAS through evolving mutations against the first lineage of bacterial mutants. The genomic study of E-PIASs identified a mutation in the tail-fiber protein that restored the phage’s virulence against the green mutants (Table 2). Ultimately, the bacteria shielded itself against this evolved phage by generating a stable brown mutant with large genomic deletions containing many genes responsible for the membrane function and integrity, including *mexY* but not the *orpM*. MexY, as a part of the MexXY-OprM efflux system, plays a key role in PIAS adsorption. We confirmed this using knock-out mutants derived from *P. aeruginosa* strain PA01 in spotting and adsorption assays. Moreover, the *orpM* knock-out mutant was also resistant to phage infection indicating the essential role of OrpM in phage-host interaction. In contrast, the disruption of the *galU* gene coding for protein involved in the LPS core region biosynthesis had no apparent effect on the phage infection process. Consequently, PIAS phage uses the MexXY with OprM as a receptor to initiate infection. The MexXY-OprM receptor has also been suggested in the case of adsorption of the OMKO1 phage from the *Myoviridae family* (Gurney et al., 2020). The brown color in the mutants is attributed to the deletion of the *hmgA* gene, causing accumulation of homogentisic acid (Rodríguez-Rojas et al., 2009). The deleted region also has many other genes necessary for bacterial defense, pathogenesis membrane integrity, transport, *quorum sensing*, and antibiotic resistance (Supplementary Table 2). Chromosomal DNA deletion-producing pigment mutants has been studied (Tanji et al., 2008; Shen et al., 2018). According to these studies, after phage infection, random brown mutants with chromosomal deletion and a white mutant with a single nucleotide variation in the LPS gene prevented phage adsorption. In Le et al. (2014), the authors also stated that the brown mutants formed spontaneously before phage infection. However, we found that no pigment mutant appeared without phage infection. Large genome deletions (50–600 kbp) have also been detected when *P. aeruginosa* is exposed to certain antibiotics such as ceftazidime and meropenem (Cabot et al., 2016, 2018; Sanz-García et al., 2018). These deletions included the *galU* gene and caused resistance to some antibiotics. In a previous study, a single *galU* mutant was shown to have increased ceftazidime MIC value, which was linked to decreased membrane permeability (Alvarez-Ortega et al., 2010). The *galU* gene is missing in our brown mutants, as well (Supplementary Table 2, GO:0006011) and extended genome deletions, including many membrane-related genes, could be identified up- and downstream of this gene. Our bile acid permeability study showed the brown mutants to have significantly increased the membrane permeability as compared to that of the wild-type strain (Supplementary Figure 1). However, in our green mutants, no point mutation could be identified in the *galU* gene. Thus the decreased MIC of ceftazidime (Table 3) in the mutants might have multiple reasons including the mutation/elimination of genes responsible for the membrane integrity. Notably, in our study, no antibiotics, but only phages were used when large genomic deletions took place. The other difference in our study from previous findings is that the PIAS uses a different receptor - MexXY-OprM - to initiate the infection. Interestingly, previous studies with phages PaP1 and PaoP5 revealed similar genomic deletion patterns in the bacterial host (Shen et al., 2018). Our study compared the genomic deletions in their host mutants generated by PIAS and PaoP5 (Figure 4A). We found that the mutants had similar deleted regions that clogged phage infection. One important difference in our study was that PaoP5 was adsorbed to the LPS, whereas PIAS interacted with MexXY-OprM as the phage receptor of bacteria. It is also important to note that phage-provoked chromosomal deletions are not limited solely to PAK-P1-like phages. Another group of *Podoviridae* phages, Ab31, Ab27, and Ab09 also gives rise to mutants with genomic deletions (Latino et al., 2014, 2019). However, the genomes of these phages (∼45 Kb) are much smaller than are those of PAK-P1-like phages (∼93 Kb). Thus, further studies are required to identify the genera to which both Ab31 and Ab27 belong and any underlying functional similarities in how these phages infect their hosts and induce deletions. In our study, we also observed phage-induced chromosomal deletion by another phage isolate, PAPSZ1, a broad-host-range phage closely related to PIAS (Figure 2A) that could also induce the formation of brown mutants from various clinical strains. However, after PAPSZ1 infection, the green mutant did not show any significant drug sensitivity compared to the wild type. We believe that PAPSZ1 uses an alternative receptor to initiate infection (to be published elsewhere). The formation of bacterial mutants with different colony morphology following phage infection has been studied (see above). However, there have been no published studies concerning the interaction between genomic deletion and restoration of antibiotic resistance. Furthermore, the current study provides insight into the short-term bacterial and phage coevolution both *in vitro* and *in vivo*.

### Compromised RND Multidrug Efflux System

*Pseudomonas aeruginosa* mainly neutralizes the action of antibiotics by reducing the permeability of its outer membrane and efficiently excluding drug molecules through efflux pumps (Lambert, 2002; Riou et al., 2016; Cunrath et al., 2019). Most common clinical resistance in *P. aeruginosa* is associated with mutations affecting efflux pumps (Kievit et al., 2001; Laohavaleeson et al., 2008; Ohene-Agyei et al., 2012; Ozer et al., 2012; Guénard et al., 2014; Singh et al., 2017; Serra et al., 2019). Evidence shows that the membrane-associated peptidoglycan and a major facilitator superfamily (MFS) transporter can also contribute to antibiotic resistance (Borisova et al., 2014; Sharma et al., 2017). Efflux pumps are vital for bacteria to overcome the current era of antibiotics. This significance has attracted them as novel drug targets, and many new efflux pump inhibitors have been developed (Lamut et al., 2019). Meanwhile, PIAS can directly utilize the components of an efflux pump for infection. This will induce a race between bacterial fitness/antibiotic sensitivity and phage resistance. Of the various RND type efflux pumps, MexAB-OprM, MexCD-OprJ, MexEF-OprN, and MexXY-OprM are extremely important. The MexXY-OprM RND comprises an outer membrane porin (OprM), a cytoplasmic-membrane antiporter (MexY), and a periplasmic membrane fusion protein that joins the membrane-associated components together (MexX) (Daury et al., 2016). This system, together with OprM, contributes to the intrinsic drug-resistance mechanism in MDR *P. aeruginosa*. In this study, the mutant colonies displayed changes (SNPs or deletions) in the *mexY* gene, potentially changing the function of the efflux system. The brown mutant showed more severe mutations, with complete loss of genes such as the *mexY* efflux transporter gene, the *mexX* precursor gene, and the regulatory gene *mexZ*. Previous studies have shown that loss of *mexY* caused sensitivity to drugs of different classes (Masuda et al., 2000). PIAS-induced mutation and subsequent deletion may interfere with the function of the efflux pump, making previous MDR clinical strains sensitive to different drug classes. Aminoglycosides are an important class of drugs in treating *P. aeruginosa* infections. Aminoglycoside resistance is widespread among CF patients. Overexpression and mutation in *mexXY* have been reported in *P. aeruginosa* isolated from CF patients (Laohavaleeson et al., 2008; Singh et al., 2017; López-Causapé et al., 2018b). Noticeably, in the PIAS induced green and brown mutants, the MIC of gentamicin decreased. In *P. aeruginosa, glpT* transporter mutation can trigger FSF resistance (Castañeda-García et al., 2009). This mutation was absent in studied wild-type strains. When *P. aeruginosa* was exposed to ceftazidime for a longer time, large region of the genome (55–600 kbp) was deleted (Sanz-García et al., 2018). However, this deletion followed by SNPs increased the MIC of ceftazidime in the mutants. In contrast to this study, in our case, the phage induced mutant had lowered MIC for ceftazidime, which might have multiple genetic reasons. An earlier study has shown that the outer peptidoglycan layer can also contribute to resistance (Borisova et al., 2014). In the PIAS-provoked bacterial mutants, we found the MIC of FSF to be lower than in the mother strains. Phage-provoked green mutants of PA16 had multiple minor deletions and SNPs in membrane-related genes that might affect the mutants’ FSF resistance. The exact mechanism underlying the FSF resistance remains unclear. We selected FSF for combinational therapy, as all the mutants became at least 5× more sensitive to FSF than were the clinical wild-type cell lines. The ability of PIAS to utilize efflux pumps as a receptor and induce deletion will qualify it as a novel phage. Thus, phage selection will be critical in using the full potential of such PIAS-like phages to combat MDR bacteria.

### *In vivo* and *in vitro* Antibiotic and Phage Combination Therapy

Though there have been ground-breaking *in vivo* studies on the efficiency of phage therapy, few recent studies have evaluated the efficiency of combinational therapy with antibiotics and phages in treating bacterial infection. A recent study of *in vivo* combination therapy with OMKO1 phage (which also utilizes efflux pumps for infection) and erythromycin showed increased sensitivity in a wax moth (*Galleria mellonella*) larvae model (Burmeister et al., 2020). For PIAS, it is vital to study how *P. aeruginosa* will respond in an environment where both the phage and antibiotics are present. During infection and the subsequent phage treatment, the actual MOI values depend strongly on the infection level, the applied phages doses, the method of administration, and the quantitative tissue distribution. We used combined phage and antibiotic therapy to challenge clinical strains of *P. aeruginosa*. The selected clinical strains were infected *in vitro* with MOI 1 PIAS and with different classes of antibiotics against which the strains were resistant. Combination treatment completely eliminated the MDR-resistant strains, and we observed no growth after 48 h incubation. This elimination may be provoked by the combined effect of the antibiotics and the phages, with the antibiotics blocking the growth of mutants and the phages killing the non-mutants. Though the way in which these gene mutations and subsequent deletions were induced at the molecular level remains unclear, the phages clearly play an active role in the process. The results of *in vivo* experiments showed that PIAS in combination with antibiotics could fight *P. aeruginosa* infection in mice. Several studies have recently shown that phage therapy can treat *P. aeruginosa* infection in animal models (Beeton et al., 2015; Cafora et al., 2019).

**FIGURE 7:**
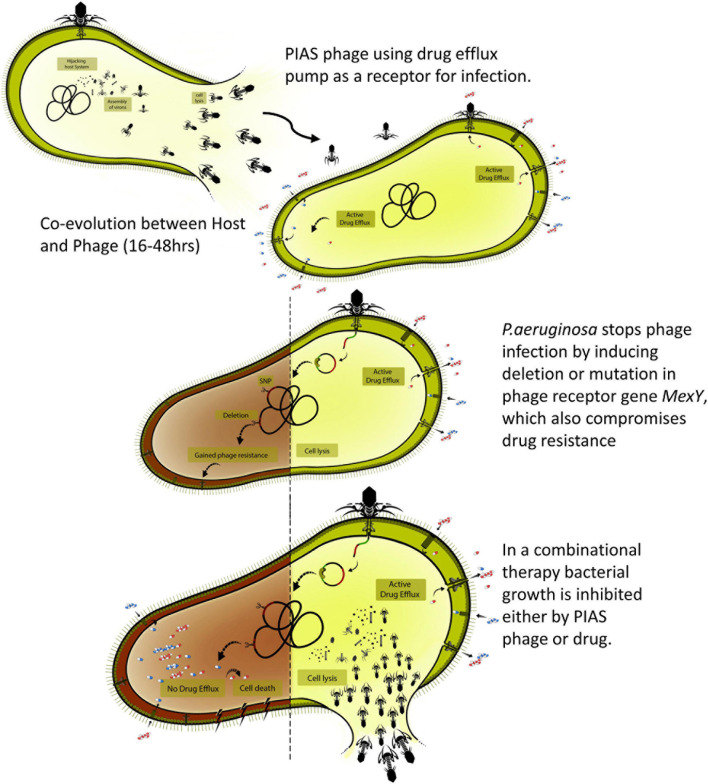
Overview of the phage-infection-provoked coevolutionary process leading to phage resistance and antibiotic sensitivity.

Nevertheless, the phage mutants isolated from the animal organs confirmed that phage–bacterial coevolution could also happen *in vivo*. As described above, we found a positive interaction between phage and antibiotic therapy, as lethality was reduced when phages and FSF were administered in combination (Figure 7). The combined action of phages and antibiotics has been demonstrated to be efficient in controlling MDR strains (Oechslin et al., 2017). As an antibacterial agent, PIAS has several properties that make it an undeniable alternative for treating MDR *P. aeruginosa*; the ability of PIAS to compromise the drug efflux mechanism, the fitness, and the pathogenicity of the target cells will add further advantages to the promising combination therapy. Combination treatment may be able to alleviate some concerns associated with phage therapeutics (Figure 7). This study shows that phages such as PIAS offer a unique window that can be exploited to eradicate MDR bacteria.

## Data Availability Statement

The datasets presented in this study can be found in online repositories. The names of the repository/repositories and accession number(s) can be found below: https://www.ncbi.nlm.nih.gov/bioproject/PRJNA720536, Bioproject: PRJNA720536; https://www.ncbi.nlm.nih.gov/, Bioproject: PRJNA57945; https://www.ncbi.nlm.nih.gov/bioproject/PRJNA722489, Bioproject: PRJNA722489.

## Ethics Statement

The animal study was reviewed and approved by Animal Welfare Committee, Permit Number: BAI/35/867-6/2019.

## Author Contributions

SKV and GR conceptualized and designed the study. ZD, GT, and EU collected and provided samples for host and phage isolation. TK and PS contributed to the genome/bioinformatic analyses. RP and TP provided the EM picture. SV, BP, and GS performed the *in vivo* animal experiments. SKV performed a number of experiments and wrote the draft of the manuscript. GR was the supervisor of the project, provided the financial background, and primarily corrected/revised the manuscript. All authors contributed to the manuscript revision, read, and approved the submitted version.

## Conflict of Interest

TK was employed by company Enviroinvest Corp and by company Biopesticide Ltd. The remaining authors declare that the research was conducted in the absence of any commercial or financial relationships that could be construed as a potential conflict of interest.

## Publisher’s Note

All claims expressed in this article are solely those of the authors and do not necessarily represent those of their affiliated organizations, or those of the publisher, the editors and the reviewers. Any product that may be evaluated in this article, or claim that may be made by its manufacturer, is not guaranteed or endorsed by the publisher.

## Footnotes

1
http://jgi.doe.gov/data-and-tools/bb-tools/
