# Supplementary figures and images for: Survival Comes at a Cost: A Coevolution of Phage and Its Host Leads to Phage Resistance and Antibiotic Sensitivity of Pseudomonas aeruginosa Multidrug Resistant Strains
Source: Front Microbiol. 2021 Dec 2;12:783722. doi: 10.3389/fmicb.2021.783722 (PMC8678094; doi:10.3389/fmicb.2021.783722)

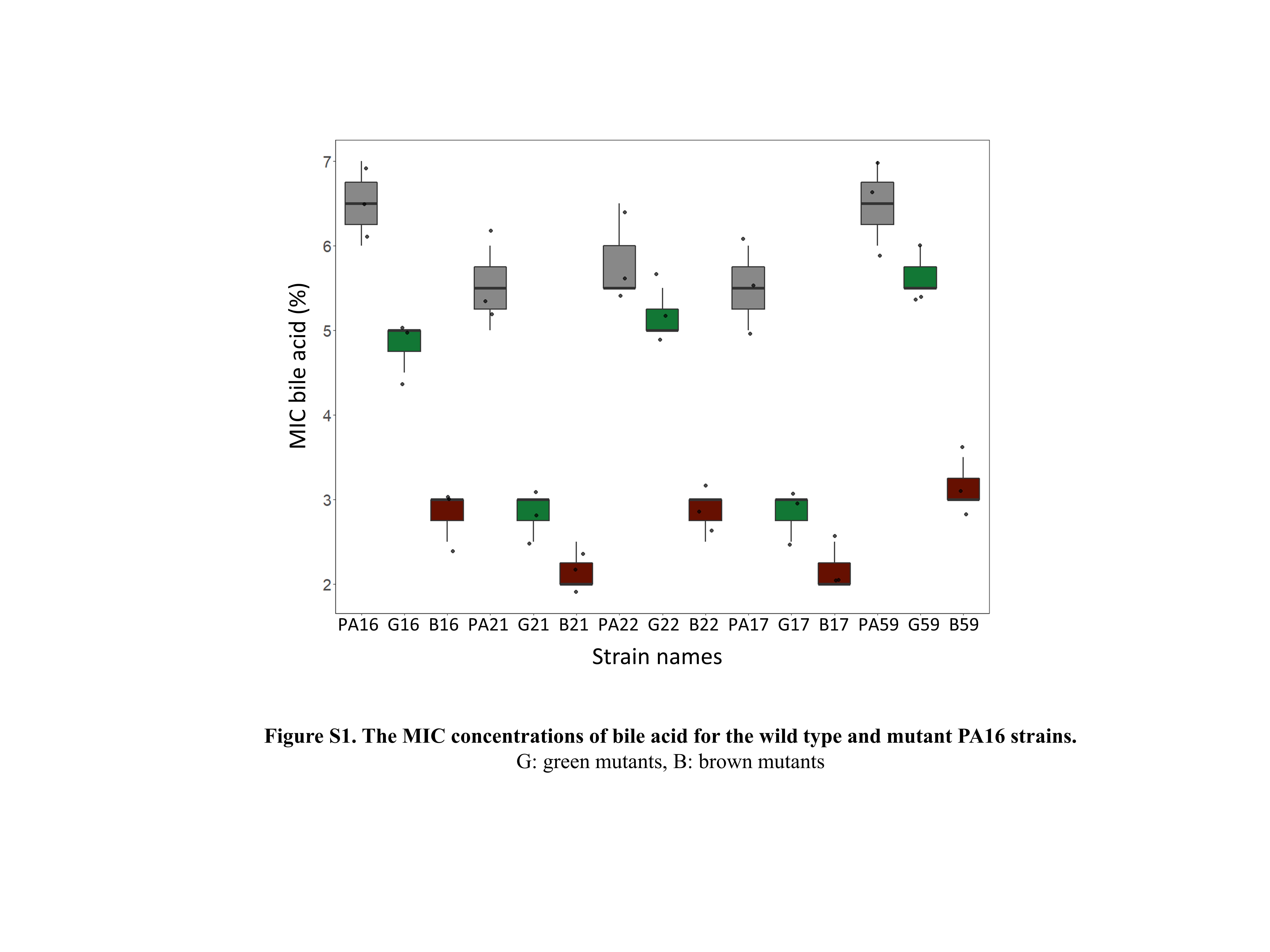

Supplement: Supplementary file 1 [file Image_1.jpg]
