# Supplementary material for: Survival Comes at a Cost: A Coevolution of Phage and Its Host Leads to Phage Resistance and Antibiotic Sensitivity of Pseudomonas aeruginosa Multidrug Resistant Strains
Source: Front Microbiol. 2021 Dec 2;12:783722. doi: 10.3389/fmicb.2021.783722 (PMC8678094; doi:10.3389/fmicb.2021.783722)
Supplement: Supplementary file 2 [file Table_1.DOCX]

**Table S1. Characteristics of the genomes of PIAS, PAPSZ1 and other *P. aeruginosa* PAK_P1-like and KPP10-like phages**

| Clade | Name | Genome length | ORFs predicted | GC content | tRNA |
| --- | --- | --- | --- | --- | --- |
| PAK_P1-like | PAK_P1 | 93398 | 181 | 49.50% | 13 |
|  | PAK_P2 | 92495 | 176 | 49.30% | 11 |
|  | PAK_P4 | 93147 | 174 | 49.30% | 13 |
|  | JG004 | 93017 | 161 | 49.30% | 12 |
|  | PaP1 | 91715 | 157 | 49.40% | 13 |
|  | PIAS | 92 397 | 181 | 49.29% | 15 |
|  | PAPSZ1 | 92 261 | 181 | 49.30% | 15 |
| KPP10-like | PAK_P3 | 88097 | 165 | 54.80% | 3 |
|  | PAK_P5 | 88789 | 164 | 54.70% | 3 |
|  | CHA_P1 | 88255 | 166 | 54.60% | 3 |
