# Supplementary material for: Survival Comes at a Cost: A Coevolution of Phage and Its Host Leads to Phage Resistance and Antibiotic Sensitivity of Pseudomonas aeruginosa Multidrug Resistant Strains
Source: Front Microbiol. 2021 Dec 2;12:783722. doi: 10.3389/fmicb.2021.783722 (PMC8678094; doi:10.3389/fmicb.2021.783722)
Supplement: Supplementary file 3 [file Table_2.docx]

**Table S2. Several selected genes deleted in a PA16 red mutants**

| **GO.ID** | **Term** |
| --- | --- |
| GO:0071453 | Cellular response to oxygen levels |
| GO:0008300 | Isoprenoid catabolic process |
| GO:0031408 | Oxylipin biosynthetic process |
| GO:0006572 | Tyrosine catabolic process |
| GO:0098743 | Cell aggregation |
| GO:0006552 | Leucine catabolic process |
| GO:0009405 | Pathogenesis |
| GO:0019441 | Tryptophan catabolic process to kynurenine |
| GO:0046247 | Terpene catabolic process |
| GO:0007155 | Cell adhesion |
| GO:0007166 | Cell surface receptor signaling pathway |
| GO:0042413 | Carnitine catabolic process |
| GO:0006559 | L-phenylalanine catabolic process |
| GO:0043711 | Pilus organization |
| GO:0061077 | Chaperone-mediated protein folding |
| GO:0044042 | Glucan metabolic process |
| GO:0006011 | UDP-glucose metabolic process |
| GO:0032329 | Serine transport |
| GO:0009439 | Cyanate metabolic process |
| GO:0090503 | RNA phosphodiester bond hydrolysis, exonucleolytic |
| GO:0005992 | Trehalose biosynthetic process |
| GO:0006303 | Double-strand break repair via nonhomologous end joining |
| GO:0071407 | Cellular response to organic cyclic compound |
| GO:0015826 | Threonine transport |
| GO:0005980 | Glycogen catabolic process |
| GO:0005978 | Glycogen biosynthetic process |
| GO:0072707 | Cellular response to sodium dodecyl sulfate |
| GO:0005975 | Carbohydrate metabolic process |
| GO:0055114 | Oxidation-reduction process |
| GO:0071555 | Cell wall organization |
