# Supplementary material for: Survival Comes at a Cost: A Coevolution of Phage and Its Host Leads to Phage Resistance and Antibiotic Sensitivity of Pseudomonas aeruginosa Multidrug Resistant Strains
Source: Front Microbiol. 2021 Dec 2;12:783722. doi: 10.3389/fmicb.2021.783722 (PMC8678094; doi:10.3389/fmicb.2021.783722)
Supplement: Supplementary file 4 [file Table_3.DOCX]

**Table S3. List of PAO1 transposon mutants used in the study**

| **Mutant** | **Defective structure(s)** |
| --- | --- |
| *ΔoprM* | Resistance-Nodulation-Cell Division(RND)-OprM precursor |
| *ΔmexY* | RND-Multidrug efflux transporter |
| *ΔgalU* | Lipopolysaccharide core region biosynthesis |
| *ΔopdO* | Pyroglutamate porin OpdO |
| *ΔsppR* | Cell surface receptor |
| *ΔlepA* | GTP-binding protein LepA |
| *ΔopdE* | membrane protein OpdE |
| *ΔgalE* | UDP-glucose 4-epimerase |
| *Δsppr* | Membrane Receptor-SppR |
| *ΔgalE* | UDP-glucose 4-epimerase |
| *ΔopdO* | pyroglutamate porin OpdO |
| *ΔcupA3* | usher CupA3 |
